# Alterations in circulating extracellular vesicles underlie social stress‐induced behaviors in mice

**DOI:** 10.1002/2211-5463.13204

**Published:** 2021-06-24

**Authors:** Shinji Sakamoto, Dania Mallah, Destynie J. Medeiros, Eisuke Dohi, Takashi Imai, Indigo V. L. Rose, Ken Matoba, Xiaolei Zhu, Atsushi Kamiya, Shin‐ichi Kano

**Affiliations:** ^1^ Department of Psychiatry and Behavioral Sciences Johns Hopkins University School of Medicine Baltimore MD USA; ^2^ Department of Psychiatry and Behavioral Neurobiology University of Alabama at Birmingham School of Medicine Birmingham AL USA

**Keywords:** exosome, extracellular vesicles, inflammation, microRNA, resilience, social defeat stress

## Abstract

Chronic stress induces peripheral and intracerebral immune changes and inflammation, contributing to neuropathology and behavioral abnormalities relevant to psychiatric disorders such as depression. Although the pathological implication of many peripheral factors such as pro‐inflammatory cytokines, hormones, and macrophages has been demonstrated, the roles of circulating extracellular vesicles (EVs) for chronic stress mechanisms remain poorly investigated. Here, we report that chronic social defeat stress (CSDS)‐induced social avoidance phenotype, assessed by a previously untested three‐chamber social approach test, can be distinguished by multiple pro‐inflammatory cytokines and EV‐associated molecular signatures in the blood. We found that the expression patterns of miRNAs distinguished the CSDS‐susceptible mice from the CSDS‐resilient mice. Social avoidance behavior scores were also estimated with good accuracy by the expression patterns of multiple EV‐associated miRNAs. We also demonstrated that EVs enriched from the CSDS‐susceptible mouse sera upregulated the production of pro‐inflammatory cytokines in the LPS‐stimulated microglia‐like cell lines. Our results indicate the role of circulating EVs and associated miRNAs in CSDS susceptibility, which may be related to pro‐inflammatory mechanisms underlying stress‐induced neurobehavioral outcomes.

## Graphical abstract

Social avoidance behaviors after chronic social defeat stress are accompanied by altered extracellular vesicles (EVs) in the blood, together with increased levels of pro‐inflammatory cytokines. EV‐associated miRNA expression patterns are correlated with social behavioral scores. In addition, EVs are shown to enhance the production of pro‐inflammatory cytokines in cultured microglia‐like cells. Thus, EVs may contribute to pro‐inflammatory responses in socially stressed mice.

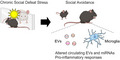

**Abbreviations**
AUC: area under the ROC curve
CCL4: C‐C motif ligand 4
CRP: C‐reactive protein
CSDS: chronic social defeat stress
CV: cross‐validation
CXCL4: C‐X‐C motif chemokine ligand 4
CXCL7: C‐X‐C motif chemokine ligand 7
EV: extracellular vesicle
FDR: false discovery rate
IFN‐α: interferon‐α
IL‐1β: interleukin 1‐β
IL‐6: interleukin 6
IL‐8: interleukin 8
LPS: lipopolysaccharides
MCP‐1: monocyte chemoattractant protein‐1
MDD: major depressive disorder
miRNAs: microRNAs
qPCR: quantitative PCR
SEM: standard error of the mean
SIR: social interaction ratio
SIT: social interaction test
SVM: support vector machine
TNF‐α: tumor necrosis factor‐α

Accumulating preclinical evidence suggests that repeated stress induces peripheral and intracerebral immune changes and inflammation, leading to neuropathology and behavioral abnormalities relevant to psychiatric symptoms such as depression and anxiety [1, 2, 3, 4, 5, 6, 7]. Consistently, altered expression of multiple inflammatory cytokines and chemokines including IL‐1β, TNF‐α, IL‐6, IL‐8, IFNα, CXCL4, CXCL7, and CCL4, and other inflammatory factors, such as CRP and MCP‐1, in the cerebrospinal fluid and/or peripheral blood are observed in patients with stress‐related psychiatric disorders such as depression, whereby high stress environments may exacerbate their perturbed regulation [8, 9, 10, 11, 12, 13, 14]. While the pathological mechanisms underlying stress‐induced behaviors mediated by circulating inflammatory cytokines, hormones, and immune cells such as macrophage have been extensively studied [1, 2, 4, 5, 6, 15, 16], other soluble factors in the peripheral blood may also play critical roles for body‐to‐brain communication of which disturbances contribute to neurobehavioral outcomes.

Extracellular vesicles (EVs) have recently gained greater attention to mediate intercellular communications by secreting various cellular components, which are transferred from donor to recipient cells in a paracrine and endocrine manner [17, 18, 19, 20, 21]. MicroRNAs (miRNAs) are small noncoding RNAs that regulate molecular expression via binding to their target mRNA 3′ untranslated region, leading to the formation of the RNA induced silencing complex, which sequesters and degrades mRNA to suppress targeting protein expression [22, 23, 24]. Recent studies demonstrate that circulating EVs could be taken up by brain cells under inflammatory conditions [19, 20, 25, 26, 27]. In addition, EV‐associated miRNAs have been shown to modulate the functions of recipient cells through modification of gene expression [20, 28, 29, 30, 31]. EV‐associated miRNAs also contribute to inflammation by triggering macrophage activation and innate immune responses [32, 33]. These results suggest that EVs and associated miRNAs may mediate inflammatory responses in the peripheral and brain immune cells.

There is a growing body of literature on the role of EVs and associated miRNAs in immune and inflammatory alterations in brain disorders [34, 35, 36, 37, 38, 39]. It has been reported that various stress paradigms induce differential expression of multiple miRNAs from peripheral origins that include serum EVs, blood NK cell EVs, and blood monocyte [35, 40, 41]. EV‐associated miRNAs (EV‐miRNAs) in the blood circulation have also been shown to reach the brain under systemic inflammation, mediating body–brain communication [42]. Nonetheless, although chronic social defeat stress (CSDS) is a widely used stress paradigm for the investigation of the neural mechanisms related to depression and anxiety and has been shown to be associated with systemic inflammation, the impact of CSDS on circulating EV‐miRNAs that potentially mediate blood‐brain communication linked to immune/inflammatory responses remains unexplored.

Here, we report CSDS‐induced differential expression of peripheral pro‐inflammatory cytokines and circulating EVmiRNAs and their relationship with social avoidance phenotypes by performing cytokine multiplex assay and miRNAs profiling. We used previously untested three‐chamber social approach test to identify ‘susceptible’ and ‘resilient’ mice to CSDS. We also demonstrate that EV‐miRNAs can distinguish CSDS ‘susceptible’ from ‘resilient’ mice. Finally, we found that circulating EVs from CSDS ‘susceptible’ mice potentiate cellular inflammatory responses. Our results highlight the importance of circulating EVs and associated molecular cargos in stress‐induced behavioral abnormalities.

## Methods

### CSDS mouse models

C57BL/6 (C57) mice were purchased from the Jackson Laboratory (Bar Harbor, ME, USA). Mice were housed in specific pathogen‐free facilities at the Johns Hopkins University. All procedures were approved by the Institutional Animal Care and Use Committee of the Johns Hopkins University and the University of Alabama at Birmingham. CSDS was performed by our published method with minor modification [43]. Briefly, aggressive male CD‐1 mice were screened out as resident aggressors and singly housed before CSDS experiments. Intruder male C57 mice were exposed to a CD‐1 aggressor for 10 min daily. After exposure, C57 mice were separated by a transparent and porous Plexiglas barrier within the home cage of the CD‐1 aggressors to enable constant sensory exposure for 24 h. CSDS was repeated with a novel CD‐1 aggressor mouse each day for 10 consecutive days. During bouts of exposure to the CD‐1 mice, hallmark behavioral signs of CSDS were observed in C57 mice including escape, submissive postures (e.g., defensive upright or supine stance), and freezing. Nonstressed control C57 mice were daily placed in a similar cage, but in the absence of exposure to aggressor CD‐1 mice.

### Three‐chamber social approach test

Social approach test was performed by our published method with minor modification [43]. Briefly, a 40 cm width × 20 cm height × 26 cm depth three‐chamber apparatus was used for the test. Both side chambers contained a plastic cage in the corner, with a plastic cup and weight on top, to prevent the subject mouse from climbing. The assay consisted of three sessions. The first session began with 10‐min habituation in the center chamber, and the second 10‐min session allowed subject mouse to explore freely all three chambers including two side chambers. Before the third session, the subject mouse was gently confined in the center chamber, while a C57 male stranger mouse (stranger) was placed in one of the two plastic cages, and an inanimate object (inanimate) was placed into another cage in the other side chamber. The inanimate objects we used were mouse toys with similar size and color as the C57 stranger mice. In the third session, the subject mouse was allowed to freely explore all three chambers for 10 min. Mouse behaviors were video‐monitored, and the trajectory of mouse ambulation was automatically determined and recorded by video tracking system, topscan 3.0 (CleverSys, Reston, VA, USA). Time spent in each chamber and sniffing each object were recorded. Based on social interaction ratio (SIR, time in chamber with stranger/time in chamber with inanimate), mice were designated as susceptible (SIR < 1.0) or resilient (SIR ≥ 1.0). Social sniffing was scored as the sum of nose‐to‐nose sniffing (sniffing or snout contact with the head/neck/mouth area) and nose‐to‐tail sniffing (sniffing or snout contact with the tail area). The heat maps were generated by ethovision xt 11.0 (Noldus, Leesburg, VA, USA).

### Multiplex profiling assay for serum cytokines

According to the manufacturer's protocol, 24 h after 10 days CSDS followed by social interaction test (SIT), cytokine, and chemokine expressions in serum at the protein level were measured by conducting multiplex profiling assays (Meso Scale Diagnostics, Rockville, MD, USA).

### EV enrichment and characterization

Extracellular vesicles were enriched using the protocol adapted from published methods with minor modifications [16, 44, 45]. Briefly, serum was collected and centrifuged at 2000 ***g*** for 30 min at 4 °C to remove cell debris. Cleared supernatants were processed to enrich EVs using Total Exosome Isolation Kit (cat# 4478360; Thermo Fisher Scientific, Waltham, MA, USA) according to the manufacturer's protocol. Following mixing and 30‐min incubation with the reagents on ice, samples were centrifuged at 10 000 ***g*** for 10 min. Pellets were resuspended in PBS and stored at 4 °C for up to 1 week.

### Transmission electron microscopy

Freshly prepared EV samples were adsorbed onto carbon‐coated/palladium grids and negatively stained with 2% (w/v) uranyl acetate. EVs were visualized under a Tecnai Spirit T12 Transmission Electron Microscope, Thermo Fisher Scientific (formerly FEI), Waltham, MA, USA.

### Nanoparticle tracking assay (NTA)

Single particles were detected with a nanosight ns 300 and nanosight nta software (ver. 3.4) (Malvern Panalytical, Malvern, Worcestershire, UK). The samples with vesicle concentrations of 10^7^ to 10^8^ were used for size distribution and concentration analyses. Raw concentration data were converted to the concentration in sera considering the dilution factor.

### Isolation of microglia‐enriched CD11b^+^ cells

Microglia‐enriched CD11b^+^ cells were isolated from the prefrontal cortex (PFC) of mice as we have previously described [43]. Briefly, mice were deeply anesthetized with isoflurane, and cardiac perfusions were performed. PFC was defined by landmarks and neuroanatomical nomenclature in the atlas of Franklin and Paxinos [46]. PFC: anteroposterior (AP): +2.57 to +1.53 mm, mediolateral (ML): ±2.75 mm from bregma, dorsoventral (DV): −1.75 to −3.05 mm from the dura according to the atlas. Bi‐lateral PFC was rapidly dissected on icy plate, minced in HBSS (Sigma‐Aldrich, St. Louis, MO, USA), and dissociated with neural tissue dissociation kits (MACS Miltenyi Biotec, Auburn, CA, USA). After passing through a 70‐μm cell strainer, homogenates were centrifuged at 300 ***g*** for 10 min. Supernatants were removed, cell pellets were resuspended, and myelin was removed using Myelin Removal Beads II (MACS Miltenyi Biotec). Myelin‐removed cell pellets were resuspended and incubated with CD11b MicroBeads (MACS Miltenyi Biotec) for 15 min, loaded on LS columns, and separated on a quadroMACS magnet. CD11b^+^ cells were flushed out, washed, and resuspended in sterile HBSS (Sigma‐Aldrich). Isolated microglia‐enriched CD11b^+^ cells were immediately used for further assays.

### RNA extraction and real‐time qPCR analysis

Isolated microglia‐enriched CD11b^+^ cells were washed three times with PBS, and total RNA was isolated using the RNeasy Mini Kit (Qiagen, Germantown, MD, USA) according to the manufacturer's guidelines. RNA concentrations were determined using a NanoDrop ND 1000 spectrophotometer (Thermo Fisher Scientific, Wilmington, DE, USA). Expression levels of TNF‐α, IL‐6, and IL‐1β mRNA were confirmed by real‐time (RT)‐qPCR analysis. In brief, cDNA synthesis was performed using SuperScript® III CellsDirect™ cDNA Synthesis Kit (Life Technologies Corporation, Grand Island, NY, USA) from total RNA in the range of 10–100 ng. Real‐time PCR contained diluted cDNA from the synthesis reaction and 200 nm specific forward and reverse TaqMan primers specific to targeted cytokine (Assay IDs for TNF‐α, IL‐6, and IL‐1β are Mm00443260_g1, Mm00446190_m1, and Mm00434228_m1, respectively) (Applied Biosystems, Foster City, CA, USA). Primers for GAPDH were used to normalize the expression data. The real‐time PCR and measurement were carried out with Applied Biosystems PRISM 7900 HT. PCR conditions were as follows: 50 °C, 2 min; 95 °C, 10 min; 40 cycles of 95 °C, 15 s and 60 °C, 1 min, including a dissociation curve at the last step to verify single amplicon in the reaction. Quantification was performed using the Δ*C*_t_ method ($2^{‐\Delta \Delta C_{t}}$). Data were normalized to GAPDH.

### Western blotting

Prefrontal cortex tissue or cell lysates were prepared with RIPA buffer and separated on 4–12% NuPAGE Bis‐Tris Mini Gels (Thermo Fisher Scientific, Waltham, MA, USA), followed by transfer to PVDF membrane (Millipore, Burlington, MA, USA) following a standard protocol. After blocking in 5% skim milk/PBS‐T, membranes were incubated with the primary antibody overnight at 4 °C and then incubated with the secondary antibody for 1 h at room temperature. Target‐specific bands were visualized by ECL substrate (Thermo Fisher Scientific, Waltham, MA, USA) and imaged with ImageQuant LAS 4000 (GE Healthcare, Chicago, IL, USA). The following primary antibodies and corresponding HRP‐conjugated secondary antibodies were used: Iba1 (1 : 500, 016‐20001, Wako Chemicals USA, Inc., Richmond, VA, USA), β‐tubulin (1 : 4000; Sigma‐Aldrich), CD9 (1 : 1000; Abcam, RRID:_10561589, Cambridge, UK), Alix (1 : 1000; Abcam, RRID:_2754981), and Calnexin (1 : 1000; Enzo Life Science, RRID:_1061834, Farmingdale, NY, USA).

### Cell culture

BV2 cells were maintained in DMEM/F12 supplemented with 15% FBS and penicillin/streptomycin (all from Thermo Fisher Scientific, Waltham, MA, USA). EV‐rich fractions (10 µg) were added to BV2 cell culture (5 × 10^4^ cell/100 μL medium per well in 96‐well culture plate) in the presence or absence of lipopolysaccharides (LPS, 100 ng·mL^−1^; Sigma‐Aldrich), and the culture supernatants were collected 6 h later. The levels of pro‐inflammatory cytokines, including TNF‐α (cat # MTA00B; R&D Systems, Minneapolis, MN, USA), IL‐1β (cat # MLB00C; R&D Systems), and IL‐6 (cat # M6000B, R&D Systems), were measured with commercially available ELISA kits according to manufacturer's protocols.

### miRNA profiling

miRNAs were collected from EV fractions using miRNeasy mini kit (cat# 217004; Qiagen) following the manufacturer's instructions. Then, miRNA expression profiling was conducted using TaqMan™ OpenArray™ Rodent MicroRNA Panel (#4470188; Thermo Fisher Scientific, Waltham, MA, USA) on QuantStudio™ 12K Flex Real‐Time PCR system (Thermo Fisher Scientific, Waltham, MA, USA). Averaged *C*_q_ value for controls (U6 snRNA) on will be subtracted from that for each miRNA to calculate Δ*C*_q_ values on each plate.

### Data analyses

#### Support vector machine model

A support vector machine (SVM) model is a method in supervised machine learning and was used to predict the CSDS‐susceptible and CSDS‐resilient mice using the expression data of circulating EV‐miRNAs (Δ*C*_q_ values). SVM models are used for both classification and regression problems, essentially creating a line or plane that separates data into classes. Such models uniquely find the best line or plane separator that will have the maximal margin from both classes. In this study, we used neurominer, version 1.05 (http://proniapredictors.eu/neurominer/index.html) [47] on matlab 2018a (MathWorks Inc., Natick, MA, USA). A unique aspect of the neurominer software is its ability to use nested cross‐validation, which involves the separation of two cross‐validation schemes: an inner cross‐validation (CV1) and an outer cross‐validation (CV2). In the inner CV1 cross‐validation, features can be selected and parameters for models can be optimized. Then, these models are applied to the held‐out information in the outer CV2 cross‐validation folds. Models are trained in the CV1 cycle, and then, the best‐performing models are applied to the CV2 data. This separation of CV2 and CV1 data avoids overfitting [47]. For our model, we used a pooled CV framework, where the outer and inner cross‐validation folds will be automatically and randomly defined, and adjusted the CV settings to include two permutations and 4‐fold for both the outer cross‐validation (CV2) cycle and the inner cross‐validation (CV1) cycle. This means that the dataset will be randomly split into four CV1 folds and go into the CV1 training cycle. The program will then cycle through all of the CV2 folds and then will repeat this entire process two times (i.e., two permutations).

#### Regularized linear regression analysis

Elastic net regularization was used to select predictor miRNAs for SIR. Elastic net regularization is a statistical approach designed to select models in the context of collinearity, which produces challenges for older stepwise selection approaches [48]. Elastic net linear regression is a combination of ridge and lasso, and uses the penalties from both the lasso and ridge techniques [13, 16]. The elastic net algorithm has two parameters to be tuned. The first is a regularization parameter, lambda (λ), controlling overfitting and can be tuned via cross‐validation. When λ = 0, no shrinkage occurs, and as λ increases, coefficients are shrunk more strongly regardless of the second parameter, α. For our elastic net model, we performed 5‐fold cross‐validation (CV) to calculate the value of λ that gives the minimum mean cross‐validated error. A 5‐fold CV will randomly divide our observations into five nonoverlapping folds of approximately equal size where the first fold will be used as the validation set, and the model is then fit on the four remaining folds. We ran this model 100 times and averaged the error curves to tune our λ in order to reduce randomness [49, 50, 51, 52]. The second parameter to tune is the α parameter (between 0 and 1) where if α is equal to 0, the model corresponds to ridge, and if α is equal to 1, the model corresponds to lasso. To optimize our α parameter, we examined the averaged minimum cross‐validated error during λ tuning from 100 runs using the following α values: 0.1, 0.3, 0.5, and 0.75. We found that α set to 0.75 resulted in the smallest averaged minimum cross‐validation error and was used to generate the predictive data meaning nonsignificant coefficients were more likely to be completely eliminated from our model (Fig. S1). The analysis was conducted with glmnet on matlab 2018a (MathWorks Inc.).

### Other statistical analyses

Statistical differences among more than two groups were determined using one‐way ANOVA, or two‐way ANOVA, followed by the Bonferroni multiple comparison test. A value of *P* < 0.05 in two‐tailed test was considered statistically significant. All data are presented as the mean ± SEM. Single regression analysis and multivariable regression analysis were used to evaluate the relationships between SIR and the level of serum cytokines or EV‐miRNAs. SIR and either serum level of IL‐1β, IL‐6, and TNF‐α or EV‐miRNAs were included in variables. Regression analyses and Pearson's correlation test were performed using spss statistic software version 25.0 (SPSS Inc., Chicago, IL, USA) and matlab 2018a (MathWorks Inc.).

## Results

### CSDS susceptibility assessed by three‐chamber social approach test

**Fig. 1:**
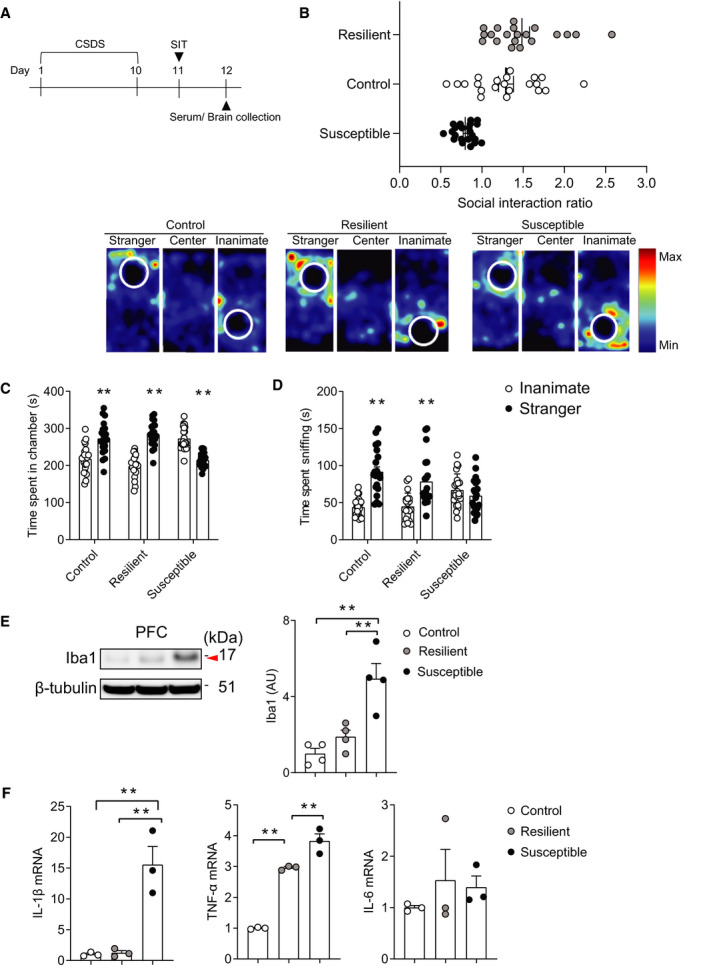
CSDS susceptibility assessed by three‐chamber social approach test. (A) Experimental timeline was shown. Eight‐week‐old C57BL/6 mice were subjected to 10 days of CSDS. Next day, three‐chamber SIT was performed, and serum or brain tissue and cell sample were subsequently collected. (B) CSDS results in a spectrum of social avoidance behavior, defined as ‘susceptible’ and ‘resilient’ phenotypes using their SIR score. Representative heat map images (lower panels) represent movements of the Control, Resilient, and Susceptible mice. (C) Susceptible mice spend significantly more time in the chamber with an inanimate object, whereas both control and resilient mice spend significantly more time in the chamber with a stranger mouse. (D) Both control and resilient mice spend significantly more time sniffing the stranger mouse, whereas there is no difference of the time of susceptible mice spend on sniffing the stranger mouse and the inanimate object. **P < 0.01, determined by two‐way ANOVA with Bonferroni multiple comparison test. (B–D) Control, *n* = 21; Resilient, *n* = 20; Susceptible, *n* = 22. (E) Western blotting for Iba1 expression in the PFC. β‐tubulin was used as loading control. Iba1 expression was increased in susceptible mice. (F) TNF‐α and IL‐1β production is increased in the microglia of the PFC of susceptible mice, compared to resilient and control mice. (E, F) **P < 0.01, determined by one‐way ANOVA with Bonferroni multiple comparison test. *n* = 3–4 per each group. Data are presented as the mean ± SEM.

Repeated exposure to social defeat stress in rodents causes behavioral abnormalities marked by social avoidance, consummatory behaviors, and anxiety‐like phenotypes [53]. While one‐chamber SIT is widely used to classify the response to the CSDS as ‘susceptible’ and ‘resilient’ subpopulation [4, 54], three‐chamber social approach test has advantages for the assessment of social behaviors [55, 56, 57, 58, 59]. Nonetheless, to our knowledge, there is no report differentiating ‘susceptible’ and ‘resilient’ behavioral phenotypes induced by CSDS using three‐chamber social approach test. Thus, we firstly examined the different social behavior phenotypes of mice subjected to CSDS by the three‐chamber social approach test (Fig. 1A). Based on previous studies using one‐chamber SIT and our data from control mice, we proposed an SIR above on or equal to 1, in which more or equal time is spent in the chamber with stranger versus in the chamber with inanimate, as the threshold for dividing defeated mice into the susceptible and resilient categories [17, 21]. While control mice showed a strong tendency to spend greater than or equal amounts of time in the chamber with stranger mouse, 22 (52.4%) out of 42 defeated mice displayed significant social avoidance behavior as indicated by their SIR (Fig. 1B). Control mice and CSDS‐resilient mice exhibited significant preference for exploring a stranger mouse relative to an inanimate object, as measured by the total amount of time spent in each chamber and sniffing each cage, whereas susceptible mice did not show preference for exploring the stranger mouse relative to the inanimate object (Fig. 1C,D). These results are highly representative of a standard social defeat experiment. We then examined the effect of CSDS on microglia, which are involved in the pathophysiological mechanisms underlying stress‐induced social abnormalities [2, 7]. After being subject to CSDS, expression of Iba1, a microglial marker, was measured by western blotting. CSDS specifically increased Iba1 expression in the PFC of the susceptible mice (*P* < 0.01, Fig. 1E). We also examined the effect of CSDS on PFC microglial cytokine expression, including IL‐1β, TNF‐α, and IL‐6, as those are reportedly increased in the cerebrospinal fluid and/or peripheral blood of patients with depression [10, 11]. We found that CSDS induced increased mRNA expression of IL‐1β and TNF‐α, but not IL‐6 in microglia‐enriched CD11b^+^ cells isolated from PFC of susceptible mice, compared with those from control mice and resilient mice (susceptible‐control; IL‐1β: *P* < 0.01; TNF‐α: *P* < 0.01; IL‐6: *P* = 0.74, susceptible‐resilient; IL‐1β: *P* < 0.01; TNF‐α: *P* < 0.01; IL‐6: *P* = 0.96, Fig. 1F). Thus, enhanced expression of IL‐1β and TNF‐α in microglia in the PFC may characterize the susceptibility to CSDS.

### CSDS‐induced elevation of serum inflammatory cytokines correlates with social avoidance phenotypes

**Fig. 2:**
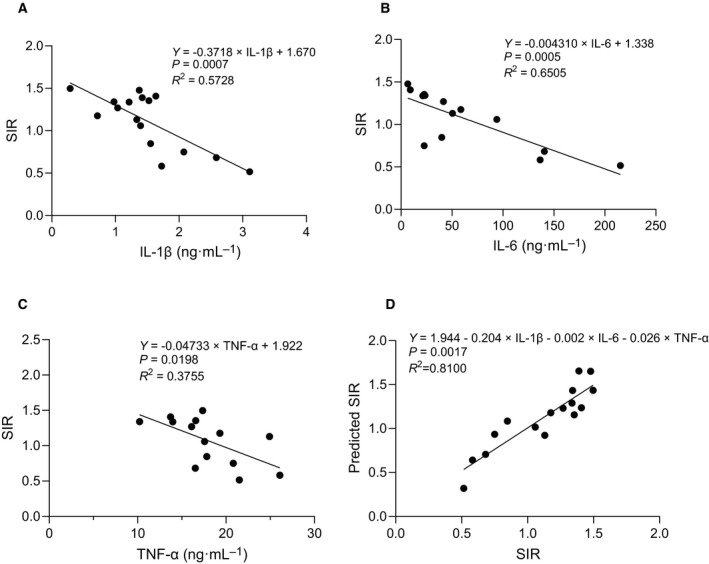
CSDS‐induced expression changes of serum pro‐inflammatory cytokines correlates with social avoidance phenotypes. (A) Single regression analysis shows an inverse correlation between IL‐1β and SIR (*R* ^2^ = 0.5728, *P* = 0.0007). *n* = 16. (B) Single regression analysis shows an inverse correlation between IL‐6 and SIR (*R* ^2^ = 0.6505, *P* = 0.0005). *n* = 14. (C) Single regression analysis shows an inverse correlation between TNF‐α and SIR (*R* ^2^ = 0.3755, *P* = 0.0198). *n* = 14. (D) Multivariable regression analysis shows a positive correlation between combination of IL‐1β, IL‐6, and TNF‐α expression and SIR (*R* ^2^ = 0.8100, *P* = 0.0017). Predicted SIR was calculated based on regression formula. *n* = 16.

Previous studies demonstrate that CSDS also increases serum levels of IL‐6 and TNF‐α expression, which contribute to social avoidance behaviors [4, 5, 60, 61]. To validate the effect of CSDS on circulating cytokine expression in the resilient and susceptible mice defined by three‐chamber social approach test, we measured serum cytokine expression in the mice subjected to CSDS by conducting multiplex profiling assays. Consistent with previous studies, expression of IL‐6 and TNF‐α in susceptible mice is higher than those in resilient and control mice (Table 1). We also observed that IL‐1β expression is elevated in susceptible mice (Table 1). Single regression analysis showed that the level of IL‐1β, IL‐6, and TNF‐α was inversely correlated with SIR, individually (IL‐1β, *R* ^2^ = 0.5728, *P* = 0.0007; IL‐6, *R* ^2^ = 0.6505, *P* = 0.0005; TNF‐α, *R* ^2^ = 0.3755, *P* = 0.0198) (Fig. 2A–C). We next wondered whether combination of measurement of these cytokines may provide better prediction of severity of social avoidance phenotypes. Compared with the results of single regression analysis, multivariable regression analysis revealed more significant correlation between combination of IL‐1β, IL‐6, and TNF‐α expression and SIR (*R* ^2^ = 0.8100, *P* = 0.0017) (Fig. 2D), suggesting that multivariable model with measurement of multiple circulating inflammatory cytokines may provide a pathological hallmark to detect stress‐induced behavioral abnormalities.

**Table 1:** The effect of CSDS on circulating cytokine expression. Mean ± SEM are shown. *P* < 0.05 are boldfaced.

Cytokines	Control (*N* = 5)	Resilient (*N* = 6)	Susceptible (*N* = 6)	*F* value	*P* value
IL‐1β (pg·mL^−1^)	1.10 ± 0.23	1.20 ± 0.13	2.21 ± 0.29	7.72	**0.0062**
IL‐6 (pg·mL^−1^)	15.13 ± 3.82	53.45 ± 10.73	110.95 ± 35.5	4.12	**0.046**
TNF‐α (pg·mL^−1^)	13.82 ± 1.31	18.90 ± 1.46	20.55 ± 1.66	4.43	**0.039**
IL‐2 (pg·mL^−1^)	3.35 ± 0.27	3.42 ± 0.31	3.94 ± 0.57	1.08	0.37
IL‐4 (pg·mL^−1^)	0.84 ± 0.15	0.49 ± 0.11	0.56 ± 0.17	1.21	0.37
IL‐5 (pg·mL^−1^)	8.45 ± 1.20	6.18 ± 0.84	7.22 ± 1.30	0.97	0.41
IL‐10 (pg·mL^−1^)	12.67 ± 2.03	12.74 ± 2.17	14.54 ± 3.35	0.14	0.87
IL‐12 (pg·mL^−1^)	12.65 ± 3.75	12.91 ± 1.92	10.16 ± 1.62	0.37	0.70
IFN‐γ (pg·mL^−1^)	1.48 ± 0.32	2.13 ± 0.30	2.50 ± 0.61	1.33	0.30
KC/GRO (pg·mL^−1^)	126.75 ± 14.22	120.26 ± 7.25	163.25 ± 47.47	0.59	0.57

### Estimation of CSDS‐induced social avoidance phenotypes by circulating EV‐miRNAs

**Fig. 3:**
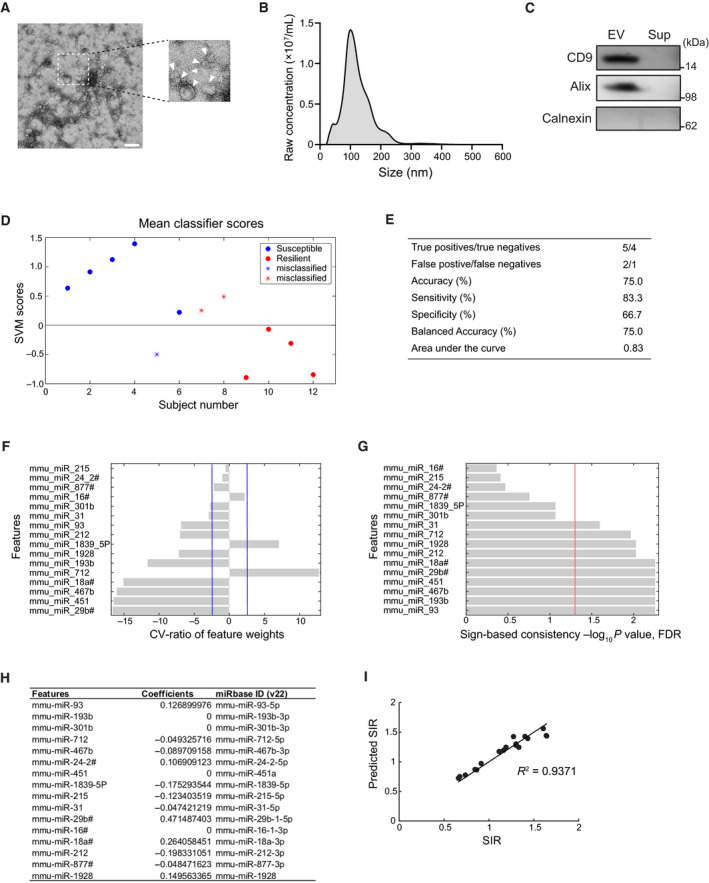
Utility of EV‐associated miRNAs as biomarkers to distinguish CSDS‐induced behavioral alterations. (A) Representative image of EV samples used in this study. EV particles are indicated by arrowheads. Scale bar, 200 nm. (B) Representative data of vesicle size distribution measured by NTA. (C) Enrichment of CD9 and Alix, exosomal protein markers, in EV fractions used in this study. Calnexin, a marker for cellular protein, was not detected in our samples. (D) Classification plot for susceptible vs. resilient mice generated by a SVM model. *n* = 6 mice per group. (E) Performance metrics of the susceptible vs. resilient SVM classifier. (F, G) Predictive signatures underlying EV‐miRNA‐based model generated with SVM. The stability of predictive pattern elements was evaluated using cross‐validation ratio mapping (F), and the significance of predictive features was assessed by means of sign‐based consistency mapping (G). Blue lines indicate the CV‐ratio threshold (CV‐ratio = ±3), and a red line indicates *P* = 0.05 (FDR), respectively. (H) miRNA predictors of SIR scores and their penalized coefficients from elastic net model. *n* = 18 mice, including six susceptible, six resilient, and six control mice. (I) Plot showing a high correlation between predicted SIR scores and observed SIR scores (*R* ^2^ = 0.9371, *P* = 4.92e‐11; Pearson's correlation test).

Previous human studies showed that blood EV miRNAs of patients with major depressive disorder (MDD) differed from those of controls and that EVs from MDD patients altered normal mouse behaviors in forced swim, tail suspension, and novelty suppressed feeding tests [39]. Thus, we examined the serum EV miRNA profiles in mice after CSDS using multiplex qPCR array. Successful collection of EVs containing exosomes from serum samples of CSDS mice was confirmed by the size and morphological analysis of vesicles assessed by transmission electron microscopy, nanoparticle tracking assay, and western blotting for EV‐enriched proteins (Fig. 3A–C). There was no significant difference in EV size and concentration between the susceptible and resilient mice (Fig. S2). Among 408 targets on the panel, 24 targets were detected in all the samples (*n* = 6, susceptible; *n* = 6, resilient; *n* = 6, control). With recent annotation data from miRbase (ver. 22), eight targets turned out to be non‐miRNA RNAs. There were no differentially expressed miRNAs in pairwise comparison (susceptible vs. resilient) among these 16 miRNAs (Table S1). We also tested if the CSDS susceptible and resilient mice could be distinguished by the patterns of EV‐miRNA expression. To this end, we first tested if EV‐miRNA data could classify susceptible from resilient mice using a SVM model (Fig. 3D–G). The analysis showed that EV‐miRNAs discriminate susceptible mice from resilient mice with an area under curve (AUC) of 0.83 (Fig. 3D,E). Ten miRNAs were determined as significant predictors by sign‐based consistency mapping [*P* < 0.05 for false discovery rate (FDR)] (Fig. 3F,G). We also performed elastic net regularized linear regression analysis using 16 miRNA data to predict SIR. The 12 miRNAs (miR‐93‐5p, miR‐712‐5p, miR‐467b‐3p, miR‐24‐2‐5p, miR‐1839‐5p, miR‐215‐5p, miR‐31‐5p, miR‐29b‐1‐5p, miR‐18a‐3p, miR‐212‐3p, miR‐877‐3p, miR‐1928) were identified as significant features to predict SIR scores (Fig. 3H). The predicted SIR scores were highly correlated with the observed SIR scores (*R* ^2^ = 0.9371, *P* = 4.92e‐11) (Fig. 3I). Among these significant predictor EV‐miRNAs (Fig. 3G,H), miR‐31‐5p, miR‐712‐5p, miR‐212‐3p, miR‐451a, miR‐467b‐3p, miR‐193b‐3p, and miR‐93‐5p were previously reported to regulate pro‐ or anti‐inflammatory responses (Table 2).

**Table 2:** Predictor miRNAs whose functions are involved in inflammatory responses.

miRNA	Pro‐/anti‐inflammatory	Effects on IL‐1 β	Effects on IL‐6	Effects on TNF‐α	Other effects	References
miR‐31‐5p	Pro‐inflammatory	↑	↑	↑		[74, 75]
miR‐712‐5p	Pro‐inflammatory, Anti‐inflammatory	a	↓	↑, ↓		[76, 77]
miR‐212‐3p	Anti‐inflammatory	a	↓	↓		[78]
miR‐451a	Anti‐inflammatory	↓	a	↓		[79]
miR‐467b‐3p	Anti‐inflammatory	↓	↓	↓		[80, 81]
miR‐193b‐3p	Anti‐inflammatory	↓	↓	↓		[82]
miR‐93‐5p	Anti‐inflammatory	↓	↓	↓		[83, 84]

^a^ Not reported.

### Upregulation of cytokines production in BV2 cells treated with serum EVs from CSDS susceptible mice

**Fig. 4:**
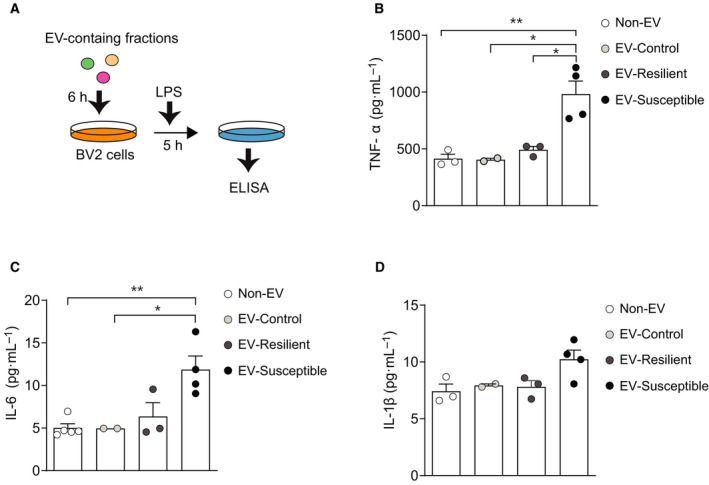
Circulating EVs collected from CSDS susceptible mice induces pro‐inflammatory cytokine production in BV2 cells. (A) Schematic diagram of assessment of cytokine production in the BV2 cells treated with EV‐containing fractions collected from susceptible mice. Exposure to EVs from susceptible mice enhanced production of (B) TNF‐α, (C) IL‐6, and (D) IL‐1β in LPS‐treated BV‐2 cells. *P < 0.05, **P < 0.01 determined by one‐way ANOVA with *post hoc* Bonferroni test. *n* = 2–5 per each condition. All data are presented as the mean ± SEM.

We next examined whether circulating EVs in the susceptible mice have a differential immunomodulatory role, compared with those from the control and resilient mice (Fig. 4A). Microglia‐like cell lines, BV2 cells, were treated with EV‐rich fractions from susceptible, resilient, and control mice for 6 h, which was started 1 h before administration of LPS (100 ng·mL^−1^), followed by measurement of IL‐1β, TNF‐α, and IL‐6 protein expression. We found that treatment with EVs from susceptible mice increased TNF‐α and IL‐6 production, compared to those from other conditions (Fig. 4B,C). There is also a trend in an increase of IL‐1β expression by treatment with EVs from susceptible mice (Fig. 4D). These results suggest that CSDS affects immune modulatory property of circulating EVs, which may contribute to inflammatory mechanisms underlying stress‐induced social avoidance phenotypes.

## Discussion

In this study, we have demonstrated that miRNAs associated with circulating EVs in the blood reflect stress‐induced behavioral alterations in mice. We have also found that EVs from stress susceptible mice potentiate pro‐inflammatory responses of microglia‐like cells. Notably, multiple miRNAs predicting stress susceptibility have been reported to be linked to inflammation. Thus, our findings suggest that chronic stress in mice induces systemic pro‐inflammatory changes both at the levels of cytokines and circulating EV miRNAs.

An increasing body of evidence suggests that EVs and their associated miRNAs are involved in the modulation of immune responses by cultured cells under various inflammatory conditions [62, 63, 64]. More recently, it has been shown that stress‐induced systemic inflammation results in the generation of EVs enriched with pro‐inflammatory miRNAs [35, 40]. These results suggest that CSDS‐induced alteration in immune modulatory property of circulating EVs in the susceptible mice may affect stress‐evoked inflammatory response of brain immune cells, which may in turn contribute to neurobehavioral consequences. A previous report showed that circulating EVs reach brain cells in the parenchyma during systemic inflammation [42]. In addition, monocytes and macrophages are known to uptake circulating EVs *in vivo* [65, 66, 67]. Nonetheless, there is currently no experimental evidence about whether circulating EVs modify behaviors in response to CSDS. Although our results suggest that circulating EVs from the CSDS‐susceptible mice elevate pro‐inflammatory responses of microglia‐like cells, it remains elusive whether circulating EVs contribute to microglia activation *in vivo*. Multiple circulating EV‐miRNAs that predict CSDS‐induced behavioral changes were previously reported to modulate the production of pro‐inflammatory cytokines, such as IL‐1β, TNF‐α, and IL‐6. Thus, these miRNAs may underlie pro‐inflammatory effects of circulating EVs from CSDS‐susceptible mice. These outstanding questions warrant further investigation.

We utilized the three‐chamber social approach test to differentiate ‘susceptible’ and ‘resilient’ mice as the response to CSDS. Compared with one‐chamber SIT that classically used for evaluating stress susceptibility, three‐chamber social approach test has advantages for the assessment of social behaviors [55, 56, 57, 58, 59]. While the social interaction is evaluated by encountering time of the test C57 mouse to CD‐1 mouse in one‐chamber SIT [54], three‐chamber social approach test assesses the preference of ‘novel target same strain mouse vs. inanimate object’ [43, 57]. This approach represents more naturally mimic the social interaction process, evidenced by loss of sociability in normal C57 mouse when the target novel C57 mouse was replaced by a CD‐1 mouse in the three‐chamber social approach test [59]. Importantly, we confirmed that resilient and susceptible mice defined by three‐chamber social approach test displayed serum pro‐inflammatory cytokine phenotypes consistent with previous studies using one‐chamber SIT [4, 5]. In addition, three‐chamber social approach test can assess the effect of stress on social novelty preference, a process of social cognitive process. A limitation of this study is that female mice were not included in the cohort. Given that CSDS protocols for female mice have recently been established [57, 58], utility of three‐chamber social approach test in the CSDS paradigm of female mice need to be explored in future studies.

Some clinical studies reported that miRNA expression is changed in blood and peripheral blood mononuclear cells of patients with depression compared with healthy controls [14, 68, 69, 70]. Altered expression of circulating miRNAs in the blood elicited by antidepressant medication and cognitive behavioral therapy has also been reported in treatment‐responsive patients [69, 70, 71, 72]. Our analyses with a supervised machine learning and a regularized regression model suggest that multiple circulating EV miRNAs may be utilized to predict animal's social behavioral status after exposure to CSDS. Although the current models are based on a very small rodent cohort and should be regarded as preliminary, such an approach may be applicable to human studies. As a previous study in Alzheimer's disease reported the utility of plasma miRNAs as useful diagnostic biomarker [73], circulating EVs and their molecular signatures in stress‐related psychiatric disorders may serve as critical biomarkers for better assessment of disease progression and therapeutic responses.

Collectively, the results of the present study highlight the importance of circulating EVs and associated miRNAs, which may contribute to the inflammatory mechanisms underlying stress‐induced behavioral outcomes. Given that EVs mediate intracellular communication via multiple biological components, further investigation to address causal roles of miRNAs in circulating EVs for stress‐induced inflammatory processes is required for better understanding of pathological body–brain communication underlying stress‐related psychiatric disorders such as depression.

## Conflict of interest

The authors declare no conflict of interest.

## Author contributions

AK and SK designed the study. SS, DJM, TI, ED, IVLR, KM, and XZ performed the experiments. SS and DM conducted the data analysis. AK, XZ, DM, and SK wrote the manuscript.

## Supporting information

**Fig. S1.** Optimization of alpha (α) parameter for elastic net regularization. (A) Cross‐validation error curves at different α parameters. Shown are average ± error of 100 times repeated 5‐fold CV. (B) Minimum cross‐validation error for each α parameter. In our model, α = 0.75 resulted in the lowest averaged minimum cross‐validated error.
**Fig. S2.** Comparison of EV size and concentration between the susceptible and resilient groups after CSDS. No significant difference was observed in EV size and concentration between the susceptible (n = 4) and resilient (n = 3) mice after CSDS.
**Table S1.** EV‐associated miRNAs that were detected in the serum of mice after CSDS.

## Data Availability

The data are available from the corresponding author upon reasonable request.
