# Supplementary material for: Alterations in circulating extracellular vesicles underlie social stress‐induced behaviors in mice
Source: FEBS Open Bio. 2021 Jun 24;11(10):2678–92. doi: 10.1002/2211-5463.13204 (PMC8487053; doi:10.1002/2211-5463.13204)
Supplement: Supplementary file 1 — Fig. S1. Optimization of alpha (α) parameter for elastic net regularization. (A) Cross‐validation error curves at different α parameters. Shown are average ± error of 100 times repeated 5‐fold CV. (B) Minimum cross‐validation error for each α parameter. In our model, α = 0.75 resulted in the lowest averaged minimum cross‐validated error. Fig. S2. Comparison of EV size and concentration between the susceptible and resilient groups after CSDS. No significant difference was observed in EV size and concentration between the susceptible (n = 4) and resilient (n = 3) mice after CSDS. Table S1. EV‐associated miRNAs that were detected in the serum of mice after CSDS. [file FEB4-11-2678-s001.docx]

**Table S1. EV-associated miRNAs that were detected in the serum of mice after CSDS**

|  | | | |  |  |  |  |  |
| --- | --- | --- | --- | --- | --- | --- | --- | --- |
|  | Control (N=6) | | Susceptible (N=6) | | Resilient (N=6) | | | |
| miRNA (∆Cq) | mean | sem | mean | sem | mean | sem |  |  |
| miR-16-1-3p | 9.12 | 0.55 | 8.16 | 0.37 | 8.79 | 0.47 |  |  |
| miR-1839-5p | 7.54 | 0.44 | 6.57 | 0.44 | 7.01 | 0.51 |  |  |
| miR-18a-3p | 7.53 | 0.32 | 6.59 | 0.37 | 7.34 | 0.43 |  |  |
| miR-1928 | 9.80 | 0.42 | 8.29 | 0.84 | 9.44 | 0.30 |  |  |
| miR-193b-3p | 1.76 | 0.67 | -0.25 | 0.53 | 0.91 | 0.61 |  |  |
| miR-212-3p | 4.02 | 0.51 | 2.67 | 0.50 | 3.42 | 0.23 |  |  |
| miR-215-5p | 6.15 | 0.67 | 5.32 | 0.37 | 5.77 | 0.94 |  |  |
| miR-24-2-5p | 5.60 | 0.50 | 4.81 | 0.39 | 5.16 | 0.28 |  |  |
| miR-29b-1-5p | 8.51 | 0.63 | 8.47 | 0.48 | 9.39 | 0.53 |  |  |
| miR-301b-3p | 5.78 | 0.71 | 3.77 | 0.54 | 4.39 | 0.60 |  |  |
| miR-31-5p | 1.11 | 1.64 | 4.29 | 0.90 | 4.84 | 1.27 |  |  |
| miR-451a | -1.76 | 0.85 | -3.44 | 0.47 | -2.13 | 0.66 |  |  |
| miR-467b-3p | 8.32 | 0.65 | 8.14 | 0.54 | 9.53 | 0.68 |  |  |
| miR-712-5p | 4.80 | 1.36 | 6.12 | 0.46 | 5.33 | 0.60 |  |  |
| miR-877-3p | 1.34 | 0.99 | 1.23 | 0.86 | 2.05 | 0.88 |  |  |
| miR-93-5p | 2.92 | 0.62 | 0.89 | 0.33 | 1.95 | 0.71 |  |  |

**
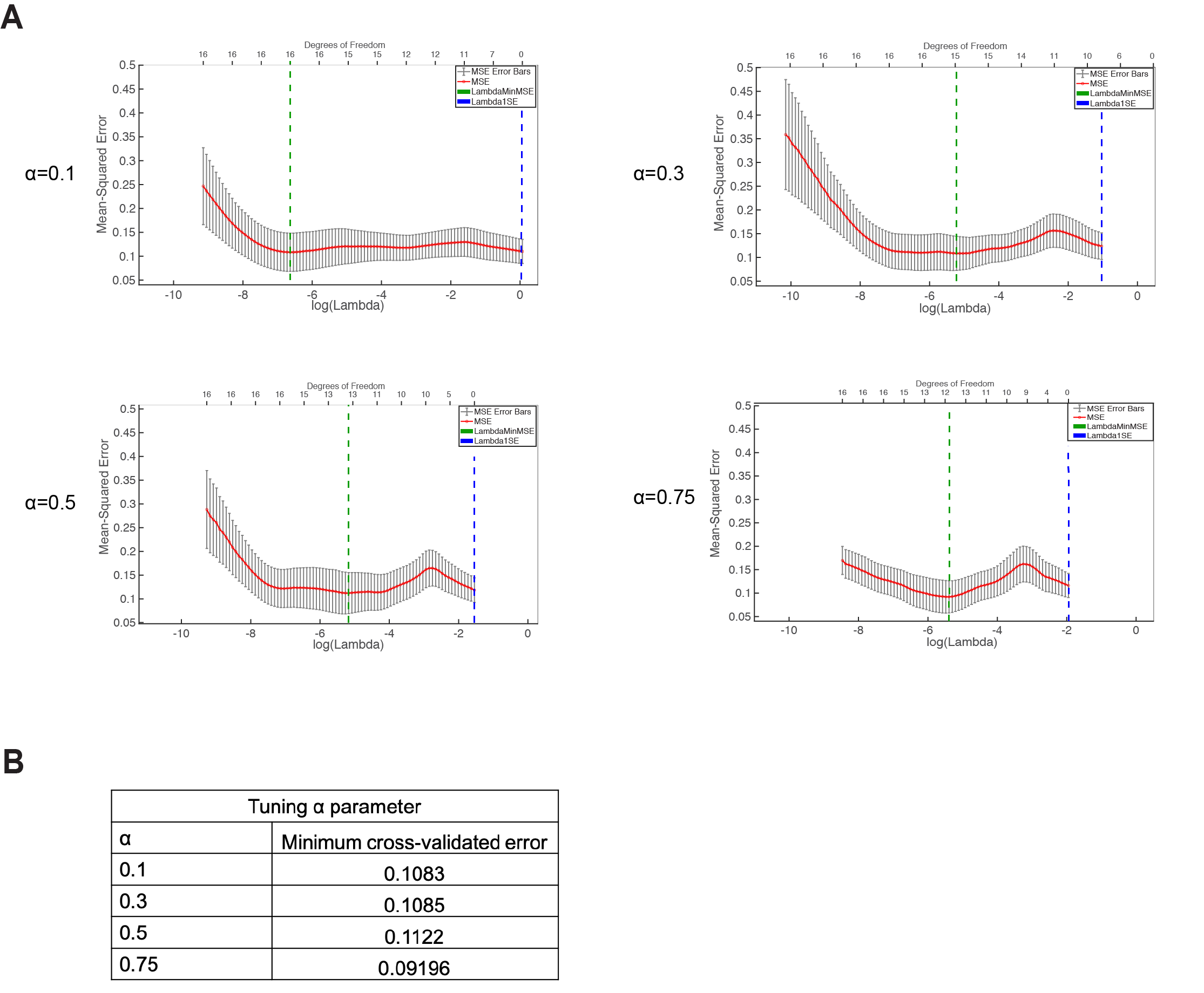
**

**Fig. S1. Optimization of alpha (α) parameter for elastic net regularization.**

(A) Cross-validation error curves at different α parameters. Shown are average ± error of 100 times repeated 5-fold CV. (B) Minimum cross-validation error for each α parameter. In our model, α =0.75 resulted in the lowest averaged minimum cross-validated error.

**
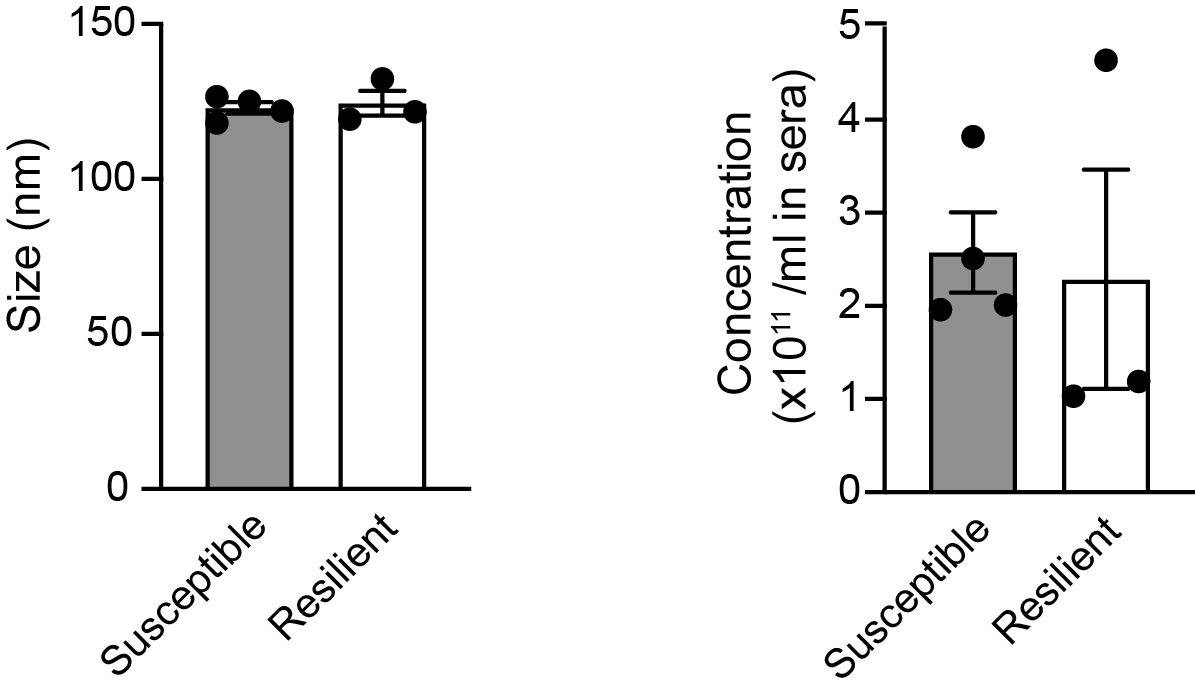
**

**Fig. S2. Comparison of EV size and concentration between the susceptible and resilient groups after CSDS.**

No significant difference was observed in EV size and concentration between the susceptible (n=4) and resilient (n=3) mice after CSDS.
